# Co-Designing Person- and Family-Centered Care for Older Adults Living With HIV: Protocol for a Community-Based Participatory Study

**DOI:** 10.2196/94520

**Published:** 2026-07-10

**Authors:** Kristina M Kokorelias, Dean Valentine, Andrew D Eaton, Marina B Wasilewski, Jill I Cameron, Stephanie Hatzifilalithis, Soo Chan Carusone, Christine L Sheppard, Jacqueline M McMillan, Rachel Landy, Adria Quigley, Hardeep Singh, Nathan M Stall, Paula A Rochon, Blessing Ojembe, Sachindri Wijekoon, Maurita T Harris, Viviane Josewski, Alice Zhabokritsky, Luxey Sirisegaram

**Affiliations:** 1 Department of Medicine, Healthy Ageing and Geriatrics Program, University Health Network, Sinai Health System, 600 University Ave, 14th Fl., Toronto, ON, M5G 1X5, Canada, 1 416-586-480 ext 4374; 2 Department of Occupational Science and Occupational Therapy, Temerty Faculty of Medicine, University of Toronto, Toronto, ON, Canada; 3 Rehabilitation Sciences Institute, Temerty Faculty of Medicine, University of Toronto, Toronto, ON, Canada; 4 Toronto Rehabiliation Sciences Institute, KITE Research Institute, Toronto, ON, Canada; 5 Jane Addams College of Social Work, University of Illinois Chicago, Chicago, IL, United States; 6 St. John’s Rehab Research Program, Sunnybrook Research Institute, Sunnybrook Health Science Centre, Toronto, ON, Canada; 7 Women's Age Lab, Women's College Hospital, Toronto, ON, Canada; 8 Department of Health Research Methodology, Evidence, and Impact, McMaster University, Hamilton, ON, Canada; 9 Factor-Inwentash Faculty of Social Work, University of Toronto, Toronto, ON, Canada; 10 Cumming School of Medicine, University of Calgary, Calgary, AB, Canada; 11 School of Health and Human Performance, Dalhousie University, Halifax, NS, Canada; 12 School of Physiotherapy, Dalhousie University, Halifax, NS, Canada; 13 Department of Medicine, University of Toronto, Toronto, ON, Canada; 14 Lunenfeld-Tanenbaum Research Institute, Sinai Health System, Toronto, ON, Canada; 15 Faculty of Social Work, University of Manitoba, Winnipeg, MB, Canada; 16 Department of Occupational Therapy, Western University, London, ON, Canada; 17 Faculty of Liberal Arts, Wilfrid Laurier University, Brantford, ON, Canada; 18 School of Nursing, University of Northern British Columbia, Prince George, BC, Canada; 19 Division of Infectious Diseases, University Health Network, Toronto, ON, Canada

**Keywords:** older adults, HIV, caregivers, chosen family, person-centered care, family-centered care, experience-based co-design, community-based participatory research, health equity, intersectionality, implementation science, qualitative research, care models, aging, health services research

## Abstract

**Background:**

Advances in antiretroviral therapy have transformed HIV into a chronic condition, leading to a growing population of adults aged 50 years and older living with HIV in Canada and globally. These individuals experience higher rates of multimorbidity, frailty, cognitive changes, and polypharmacy than their HIV-negative peers, and many rely on caregivers for emotional and practical support. Caregiving often occurs within chosen families of partners, friends, and community members, yet these caregivers remain largely unrecognized in policies that prioritize bio-legal family structures. Existing person- and family-centered care (PFCC) models in HIV focus mainly on pediatric and adolescent populations, leaving a critical gap in guidance for older adults and their diverse caregivers.

**Objective:**

This study aims to co-design Canada’s first equity-informed person- and family-centered care model tailored to older adults living with HIV and their biological, bio-legal, and chosen family caregivers. The objectives are to (1) examine experiences of accessing and providing caregiving support, including gendered and intersectional differences; (2) identify key components of a PFCC model to improve access and care experiences; and (3) develop a context-sensitive implementation strategy to support uptake across care settings.

**Methods:**

This multiphase study uses an equity-informed Experience-Based Co-Design methodology guided by the UK Design Council’s Double Diamond framework. An advisory committee of knowledge users, clinicians, and older adults living with HIV will provide ongoing input. Phase 1 involves approximately 45 semistructured interviews with older adults living with HIV, caregivers, and health care professionals, analyzed using reflexive thematic analysis. Phase 2 includes 2 co-design workshops with about 45 participants to collaboratively develop model components. Phase 3 includes 3 to 4 focus groups (approximately 30 participants) guided by the Consolidated Framework for Implementation Research to refine an implementation strategy. Equity will be operationalized using the Culturally-Competent Research Criteria for Methodological Areas and monitored using PROGRESS-Plus (place of residence, race/ethnicity, occupation, gender, religion, education, socioeconomic status, social capital, plus additional factors) indicators to support intersectional analysis.

**Results:**

The study was funded on July 17, 2025. Data collection for phase 1 is scheduled to begin in January 2027, with subsequent co-design workshops (phase 2) planned for fall 2027 and evaluation activities (phase 3) anticipated in winter-spring 2028. Completion of data analysis is expected by mid-2028, with dissemination of findings anticipated later in 2028.

**Conclusions:**

This study will generate a rigorously developed, equity-informed PFCC model grounded in lived experience. Findings will inform policy, program planning, and service delivery to better support older adults living with HIV and their diverse caregivers while offering a replicable framework for co-designing inclusive care models in other chronic or stigmatized conditions.

## Introduction

Advances in antiretroviral therapy have transformed HIV from a terminal illness into a chronic condition, resulting in a rapidly growing population of older adults aged 50 years and older living with HIV [1-8]. This demographic shift, colloquially described as the “graying of the HIV/AIDS epidemic,” has exposed significant gaps in health and social care systems that were not designed to address the intersecting realities of aging, HIV, and long-term caregiving [1]. Although people with HIV are living longer, many now experience complex and evolving health and social care needs that extend well beyond viral suppression [1-8].

Older adults living with HIV experience a higher burden of comorbidities than their HIV-negative peers, including cardiovascular disease, diabetes, osteoporosis, select cancers, and neurocognitive impairment [9,10]. They are also at increased risk of accelerated frailty and earlier onset of geriatric syndromes, driven by viral replication, especially when uncontrolled by medication in the time between conversion and treatment initiation, chronic inflammation, and long-term exposure to antiretroviral therapy, particularly older formulations that had higher levels of mitochondrial toxicity [9-12]. These biological vulnerabilities are compounded by high rates of polypharmacy, mental health challenges, and functional decline [13,14]. At the same time, many older adults living with HIV face intersecting forms of stigma and discrimination, including ageism, racism, sexism, homophobia, heteronormativity, and HIV-related stigma, which continue to shape access to and experiences of care [15-17]. Together, these factors underscore the need for integrated, geriatric- and patient-informed HIV care that spans health and social service systems. However, best-practice Canadian models for such care remain poorly defined [18].

As health needs become more complex, older adults living with HIV increasingly rely on family caregivers for emotional, physical, and practical support [19-23]. Unlike caregiving in many other chronic conditions, caregiving in HIV frequently occurs within chosen families, defined as nonbiological networks of friends, partners, and community members formed in response to stigma, social exclusion, and disrupted family relationships [19-25]. One study found that 80% of people living with HIV identified their caregivers as friends rather than biological or legal family members [26]. “Chosen family caregivers” provide critical support across the care continuum, including medication management, advocacy, navigation of fragmented systems, and assistance with daily activities [21-23]. Their unpaid labor contributes substantially to the sustainability of the Canadian health and social care system [27-29].

Despite their central role, chosen family caregivers remain largely invisible within existing health and social care policies, programs, and funding structures, which continue to privilege traditional bio-legal family models [30-39]. This lack of recognition contributes to fragmented care, increased caregiver burden, and poorer outcomes for both caregivers and older adults living with HIV [26,40-46].

Eligibility criteria for caregiver benefits and formal supports in Canada often exclude chosen family caregivers, further entrenching inequities [47]. As a result, many caregivers report unmet support needs, declining quality of life, and heightened experiences of stigma, exclusion, and emotional distress [30,48-51].

Gender further shapes caregiving experiences in important and underexplored ways. Women continue to shoulder a disproportionate share of unpaid caregiving labor and report higher levels of fatigue, emotional distress, and secondary stigma when caring for older adults living with HIV [31,52,53]. Sexual and gender minority caregivers also experience discrimination, microaggressions, and exclusion within health care systems that remain largely heteronormative and cisnormative [54-57]. While some literature has examined caregiving among Two-Spirit, Lesbian, Gay, Bisexual, Trans, Queer, and Intersex older adults more broadly, there remains limited HIV-specific, gender-informed research focused on caregiving in later life [26,58,59]. This represents a critical gap, particularly as the population of older women and gender-diverse individuals living with HIV continues to grow [60-62].

Person- and family-centered care (PFCC) has been widely promoted as an approach to improving care experiences and outcomes by actively involving patients and caregivers in decision-making, care planning, and service delivery [63,64]. Existing PFCC frameworks emphasize collaboration, recognition of family context, supportive policies, and education for patients, caregivers, and health care professionals (HCPs) [64]. However, PFCC models in HIV care have focused almost exclusively on pediatric and adolescent populations, with little attention to older adults or nontraditional family structures [64]. As a result, there is limited guidance on how PFCC can be operationalized for older adults living with HIV and their diverse caregivers across care settings.

In response to these gaps, this study aims to develop Canada’s first PFCC model tailored to the needs of diverse caregivers of older adults living with HIV. Using an equity-informed, experience-based co-design approach, we will engage older adults living with HIV, caregivers, and health and social care professionals to (1) examine experiences of accessing and providing caregiving support, including gendered differences; (2) identify key components of a PFCC model that improve access to and experiences with care; and (3) develop an implementation strategy to support uptake across care settings. By centering chosen families and addressing structural inequities, this work seeks to strengthen caregiver support, improve care experiences, and advance equity in aging and HIV care in Canada.

The findings of this study will be valuable to policymakers, health system leaders, and community-based organizations seeking to design and implement more inclusive models of care for older adults living with HIV and their caregivers. Additionally, the study has the potential to inform evidence-based policy, program planning, and funding decisions by explicitly recognizing and supporting chosen family caregivers within health and social care systems. While the research is situated in Ontario, Canada, its findings may be applicable to other international contexts with similar health system structures, aging HIV populations, and policy environments that continue to privilege bio-legal family definitions. Moreover, this study offers a rigorous, equity-informed, experience-based co-design methodological framework that can be adapted by researchers examining caregiving, aging, and chronic illness across diverse populations and care settings.

## Methods

### Study Design

This study will use a phased, multimethod approach grounded in an equity-informed Experience-Based Co-Design (EBCD) methodology [65,66], informed by prior HIV and co-design work [67]. EBCD is a participatory action research approach that meaningfully engages patients, caregivers, and HCPs as cocreators of care innovations, ensuring that the resulting models are grounded in lived experience and aligned with principles of PFCC [32,65,66]. To structure the co-design process, we will apply the UK Design Council’s Double Diamond framework, which consists of 4 iterative phases: Discover, Define, Develop, and Deliver [33]. This framework provides a transparent and replicable structure for moving from problem identification to implementation readiness. The study was codeveloped with a pre-grant advisory committee [68]. The advisory committee includes knowledge users (3 nonprofit administrators), 2 older adults living with HIV, and 5 clinicians. Members have contributed to study conceptualization, methodological decisions, and recruitment strategies. The advisory committee will meet quarterly throughout the project to provide input on study processes, support the interpretation of findings through peer debriefing, and guide integrated knowledge translation activities [34].

As co-design guidelines are still being developed [35], this study will be reported in accordance with established reporting guidelines for qualitative and participatory research. Specifically, we will follow the Standards for Reporting Qualitative Research to guide comprehensive reporting of study design, data collection, and analysis [36]. Given the participatory and co-design nature of this study, we will additionally apply the Guidance for Reporting Involvement of Patients and the Public to ensure transparent reporting of patient and community engagement throughout all phases [37]. Completed checklists will be provided as supplementary materials in related publications.

### Methodological and Equity Frameworks

An explicit equity lens guides all phases of the study to ensure inclusivity, cultural responsiveness, and attention to structural determinants of health [38,39,69-73]. Equity is operationalized using the CCRCMA (Culturally-Competent Research Criteria for Methodological Areas) framework, which informs problem formulation, recruitment, data collection, analysis, and dissemination [74]. Participant diversity will be monitored using the PROGRESS-Plus framework, capturing social determinants such as place of residence, race/ethnicity, occupation, gender, religion, education, socioeconomic status, social capital, plus additional factors [75].

Gender identity data will be collected using inclusive categories aligned with Rainbow Health Ontario recommendations [76], with options to self-describe or decline. Intersectional analysis will be applied during data interpretation to examine how overlapping social identities and systems of oppression shape caregiving experiences [77-81].

### Study Population and Recruitment

Across all study phases, we will recruit older adults living with HIV (aged ≥50 y), caregivers (including both biological/legal family and chosen family), and HCPs who provide care or support to older adults living with HIV in Ontario. Including older adults living with HIV ensures that the model is grounded in their lived experiences, needs, and priorities. To address the underrepresentation of chosen family caregivers, we will ensure that at least 50% of caregiver participants in each phase are drawn from chosen family networks. Recruiting both chosen and biological or legal family caregivers will allow us to capture a range of perspectives and examine both shared and distinct caregiving experiences, thereby strengthening the development of a PFCC model that is responsive to diverse caregiving contexts. HCPs will be included to provide insight into current care practices, challenges in supporting caregivers, and opportunities to enhance caregiver integration. Eligibility for HCP participants will require that at least 10% of their caseload consists of older adults living with HIV, ensuring adequate experience with this population.

We will use a purposive sampling matrix informed by a maximum variation strategy to ensure diversity across key characteristics [82]. Specifically, older adults living with HIV and caregivers will be recruited to reflect variation in (1) gender identity (with at least 50% male or gender-diverse participants); (2) geographic location (urban, suburban, rural, and remote regions); (3) socioeconomic status and lived experience (including low income and housing instability); (4) racial, ethnic, and linguistic backgrounds; (5) immigration and residency status (eg, newcomers); (6) sexual orientation (with at least 50% identifying as sexual minorities); and (7) age (≥50 y for older adults living with HIV). Our team has demonstrated success in recruiting across these groups in prior studies. For HCPs, in addition to gender and geographic diversity, we will ensure variation in (1) organizational setting (eg, hospitals, community-based organizations, nonprofits, and independent practices); (2) professional role (eg, frontline providers, managers, nurses, and physicians); and (3) years of experience (early-, mid-, and late-career).

Participant identification and recruitment will be conducted collaboratively with the research team, advisory committee members, community partners, and health care networks, enabling targeted outreach to underrepresented groups. Recruitment strategies will include partnerships with community organizations, outreach through social media, in-person engagement, rapport-building activities, and word-of-mouth referrals (snowball sampling). Flexible and tailored recruitment approaches will be used to enhance accessibility and inclusivity. All data collection phases and sessions will have translators available for non-English speakers.

We will implement multiple strategies to promote sustained engagement and reduce participation barriers among older adults living with HIV, caregivers, and HCPs. These include partnering with trusted community organizations, using relationship-based recruitment approaches, offering flexible scheduling (including evenings and weekends), and providing participants with a choice of interview modality (in person, by phone, or via secure virtual platforms). Participants will receive honoraria at each phase to recognize their time and contributions. We will also maintain ongoing communication (eg, reminders and check-ins) and create a supportive, inclusive study environment grounded in trauma-informed and culturally sensitive practices. To facilitate participation in virtual data collection, where applicable, participants will be offered brief, one-on-one orientation sessions on how to use the selected platform (eg, Zoom), including step-by-step guidance and troubleshooting support. Written and visual instructions will be provided in plain language. Where needed, we will offer telephone-based participation as an alternative to reduce digital access barriers. The research team has prior experience supporting participants with varying levels of digital literacy. Recognizing potential concerns related to stigma, confidentiality, and digital privacy, particularly among people living with HIV, we will use secure, institutionally approved platforms for virtual data collection and encrypted data storage. Participants will be informed about privacy safeguards, including the option to keep cameras off, use pseudonyms, and participate from a location of their choosing. Consent procedures will emphasize voluntary participation and the right to withdraw at any time. Collaboration with trusted community partners and advisory committee members will further support trust-building, cultural safety, and the acceptability of study procedures.

### Data Collection and Analysis

The study will be conducted in 3 phases.

#### Phase 1: Discovering and Defining the Problem

Phase 1 uses a qualitative descriptive design [83]. Approximately 45 semistructured interviews (15 per participant group: older adults living with HIV, caregivers, and HCPs) will be conducted, with additional interviews completed if needed to achieve thematic saturation [84]. Demographic data will be collected using the PROGRESS-Plus framework to support intersectional analyses that will examine how multiple social identities and factors, such as age, gender, ethnicity, and socioeconomic status, interact to influence experiences and outcomes [75]. Interviews will be conducted in person or virtually, audio-recorded, transcribed verbatim, and analyzed concurrently using inductive content analysis following Braun and Clarke codebook method [85] and reflexive thematic analysis techniques [86]. Analysis will follow six recursive phases: (1) data familiarization, (2) systematic coding, (3) generating initial themes, (4) reviewing and refining themes, (5) defining and naming themes, and (6) producing the report. Coding will be primarily inductive and semantic while remaining attentive to latent meanings relevant to the research aims. Coding will be conducted iteratively. A flexible codebook will be developed to support consistency across team members while allowing for ongoing refinement as new insights emerge. Regular analytic meetings will be held to discuss coding decisions, theme development, and interpretation.

A reflexive approach will be maintained throughout, with researchers documenting assumptions, positionality, and analytic decisions through memos and team discussions, consistent with Braun and Clarke emphasis on researcher subjectivity as an analytic resource [68]. The advisory committee will contribute to interpretation by reviewing preliminary themes and ensuring alignment with lived experience perspectives. Interviews will be analyzed in batches, and saturation will be considered achieved when no new codes, categories, or themes emerge across consecutive interviews within and across participant groups and when existing themes are sufficiently well-developed in terms of depth and variation [87]. The advisory committee will assist with data analysis by reviewing preliminary findings, providing interpretation from the perspective of lived experience, identifying patterns or themes that may be overlooked, and helping ensure that results are meaningful and relevant to the community. NVivo software will be used for data management and coding [88].

#### Phase 2: Co-Designing PFCC Model Components

Phase 2 employs participatory design methods through 2 iterative, hybrid co-design workshops [89,90]. Approximately 45 participants (older adults, caregivers, and HCPs) will collaboratively develop PFCC model components. Participants from phase 1 will be invited to continue, supplemented by additional recruits as needed. New participants will be recruited as needed. All participants will complete a demographic data form.

The two workshops, 2 to 3 hours each, will be held 3 to 6 weeks apart, with an optional 1-hour follow-up session to validate the prototype. Workshops will use a mix of joint and breakout-group activities to foster equitable participation, prioritize lived experience, and support candid discussion. Activities will include relationship-building exercises; storytelling and journey mapping to capture experiences and barriers; brainstorming and visual ideation (eg, mind maps and affinity diagrams) to generate potential PFCC components; prioritization exercises to identify critical elements; and prototype cocreation of care coordination strategies, educational resources, and supportive policies and procedures. Outputs from each workshop will be synthesized iteratively to inform the next session. The workshops will incorporate strategies informed by the Experience-Based Co-Design framework developed by Mulvale and colleagues [91]. Reflexive thematic analysis will be applied to transcripts, artifacts, and field notes to generate a PFCC model prototype [86]. Themes will be reviewed with the advisory committee to ensure alignment with participant perspectives and practical feasibility.

#### Phase 3: Implementation Planning

Phase 3 uses qualitative descriptive methods informed by implementation science to develop a context-sensitive implementation strategy [92]. Approximately 30 participants (~10 per group: older adults living with HIV, caregivers, and HCPs) will be recruited, prioritizing continuity from earlier phases while ensuring diverse perspectives across gender, care setting, and professional roles. Data will be collected through 3 to 4 virtual Experience Focus Group sessions, each 2 to 3 hours long, structured using the Experience Group Methodology and guided by the Consolidated Framework for Implementation Research (CFIR) [93,94]. Participants will review the draft PFCC model, identify barriers such as resource constraints, fragmented systems, or provider resistance, and propose strategies to enhance feasibility, sustainability, and scalability, including integration into community HIV programs and advocacy for caregiver recognition.

Experience Group focus groups will be guided by the CFIR [93,94]. Data will be analyzed using a combined inductive and deductive codebook thematic analysis [85], applying CFIR constructs to categorize known implementation factors and identifying emergent themes related to implementation challenges and opportunities. Initial coding will capture emergent, data-driven insights related to implementation experiences and needs, followed by mapping of codes onto relevant CFIR domains to examine known implementation determinants. Themes will be developed through an iterative and reflexive process, ensuring both conceptual depth and practical relevance. As in phase 1, analytic rigor will be supported through reflexive memoing, regular team discussions, advisory committee input, and systematic documentation of analytic decisions [95]. The expected outcome is a finalized, co-designed PFCC model with a detailed, contextually grounded implementation roadmap that can guide adoption across diverse health care settings and inform scalable interventions to support older adults living with HIV and their caregivers.

### Ethical Considerations

This study involves human participants and has received ethical approval from the Sinai Health Research Ethics Board, Ontario, Canada (approval number 25-0043-E). All participants will provide informed consent prior to participation. Consent will be obtained in writing after participants have received detailed information about the study purpose, procedures, risks, benefits, and their rights, including the right to withdraw at any time without penalty. Ongoing assent will be sought during the co-design workshops. All data will be handled in accordance with institutional and provincial privacy standards. Interview recordings will be transcribed verbatim and deidentified, with all personally identifying information removed or replaced with pseudonyms. Data will be stored on secure, password-protected institutional servers, with access restricted to authorized research team members. For virtual data collection, secure, institutionally approved platforms will be used. Participants will be reminded to join from a private location and may choose to keep cameras off or use pseudonyms to further protect their identity. Participants will receive an honorarium in recognition of their time and contributions at each phase of the study. Compensation amounts will be appropriate to the level of participation and consistent with institutional and funding guidelines.

### Materials, Protocols, and Analytic Transparency

All study materials, including interview guides, demographic questionnaires, workshop facilitation guides, coding frameworks, and analytic protocols, will be provided as supplementary materials to enable replication and adaptation in subsequent manuscripts. All publications emerging from this study will be reported using established qualitative reporting standards and EBCD methodological guidance.

## Results

This study was funded on July 17, 2025. The project is structured across 3 sequential phases: exploratory qualitative inquiry (phase 1), co-design (phase 2), and evaluation and implementation planning (phase 3). Phase 1 data collection is scheduled to begin in January 2027 and will involve qualitative interviews with older adults living with HIV, family or care partners, and health and social care providers. Phase 2, consisting of co-design workshops to collaboratively develop the care model, is planned for late 2027. Phase 3, which includes qualitative evaluation and refinement of the co-designed model, as well as development of an implementation roadmap, is anticipated from January to April 2028. Data analysis will occur iteratively following each phase, with final analytic integration expected to be completed by mid-2028. Dissemination activities, including peer-reviewed publications and knowledge translation outputs, are anticipated in late 2028.

The study is designed to generate a comprehensive understanding of barriers and facilitators to equitable caregiving support for older adults living with HIV. Outputs from phase 1 will inform the co-design process in phase 2, resulting in a prototype model of PFCC. This model is expected to include components such as inclusive caregiver recognition, care coordination strategies, tailored education, and supportive policy considerations. Phase 3 will refine the model and produce an implementation roadmap outlining strategies to support feasibility, scalability, and sustainability across care settings. Any minor modifications to recruitment or data collection procedures, if required, will be documented and justified in subsequent reports.

## Discussion

### Study Implications

This study will provide novel insights into the experiences and support needs of older adults living with HIV and their diverse caregivers, highlighting both gaps in existing services and strategies to enhance PFCC. By co-designing a PFCC model with end users and health care providers, we anticipate producing a framework that is inclusive, culturally safe, and aligned with real-world care experiences. Our findings are expected to build on previous research showing that participatory approaches and tailored interventions improve health outcomes and reduce caregiver burden while supporting more efficient health care delivery [64]. The anticipated results will likely demonstrate that integrating chosen family caregivers alongside biological and legal caregivers strengthens care coordination, improves continuity of support, and addresses inequities in access to services.

The implications of this work could extend beyond the HIV context. A co-designed, equity-informed PFCC model can serve as a blueprint for addressing the complex needs of older adults with chronic or stigmatized conditions, guiding both practice and policy. Embedding implementation science strategies ensures the model’s feasibility, scalability, and sustainability in health care settings, offering actionable recommendations for clinicians, administrators, and policymakers. By systematically incorporating end-user perspectives and intersectional social determinants of health, this approach advances inclusive and responsive health care design.

Future research should evaluate the PFCC model’s effectiveness in improving caregiver and patient outcomes, its adaptability across different health care settings, and its potential for integration into broader policy frameworks. Longitudinal studies could examine how sustained implementation impacts health care utilization, caregiver well-being, and quality of life among older adults living with HIV. Ultimately, this work lays the foundation for a person- and family-centered approach that not only addresses current gaps in HIV care but also informs the development of inclusive caregiving policies and practices in diverse health care contexts.

### Principal Results

Data collection has not yet begun but is planned to start in summer 2026. Data analysis will commence following completion of data collection. We anticipate that this study will identify key barriers and facilitators to supporting older adults living with HIV and their diverse caregivers, including systemic gaps, caregiver burden, and inequities in care access, as well as strategies that enable effective, person- and family-centered support. Co-design workshops are expected to yield a prototype PFCC model incorporating care coordination, inclusive recognition of chosen and biological caregivers, tailored education for caregivers and health care providers, and communication and policy supports. Focus groups on implementation will likely highlight practical strategies for adoption, scalability, and sustainability across Canadian care settings. Collectively, these findings will provide actionable guidance for developing equitable, culturally safe, and sustainable interventions; inform policy and practice; and offer a replicable methodological framework for researchers aiming to co-design PFCC models in diverse health care contexts.

### Limitations

This study has limitations. The research was conducted in Ontario, Canada, which may limit transferability to other jurisdictions with different health care systems or social contexts. Participation bias is possible, as individuals engaged with community networks or comfortable with virtual platforms may be overrepresented [96]. Recruitment may also be challenging due to differences in urban vs rural populations, which could also affect the representativeness of the sample. Additionally, while co-design workshops provide rich qualitative insights, translating these insights into widely adoptable interventions will require further feasibility testing.

### Comparison With Prior Work

The proposed project is rooted in existing participatory action research initiatives with older adults living with HIV [55,67,81,97-99]. A key area identified by this work is the need to center diverse caregivers (ie, biological, bio-legal, and chosen family) in future care initiatives. To build on this patient-identified gap in service and research, this project was codeveloped with a pre-grant advisory committee [67]. The research team will continue to work closely with this advisory committee throughout the duration of the project.

### Conclusions

This study addresses a critical gap in HIV and aging care by centering the experiences of older adults living with HIV and their diverse caregivers, including chosen family members. Through an equity-informed, Experience-Based Co-Design approach, we developed a PFCC model that is grounded in lived experience and responsive to the complex, intersecting needs of this population. The resulting model provides actionable guidance for inclusive care coordination, caregiver recognition, tailored education, and supportive policies. By integrating implementation science principles, the study also produces a context-sensitive roadmap to support adoption, scalability, and sustainability of the PFCC model across diverse Canadian care settings. Ultimately, this work has the potential to improve caregiver support, enhance care experiences for older adults living with HIV, and advance equity in health and social care systems. Furthermore, the methodological framework outlined in this protocol offers a replicable approach for co-designing inclusive care models for other chronic or stigmatized conditions.

## Supplementary material

10.2196/94520 Peer Review Report 1 Peer review report from Health Services Evaluation & Interventions Research 3 Committee, Canadian Institutes of Health Research (CIHR).

## Abbreviations

CCRCMA: Culturally-Competent Research Criteria for Methodological Areas
CFIR: Consolidated Framework for Implementation Research
EBCD: Experience-Based Co-Design
HCP: health care professional
PFCC: person- and family-centered care
PROGRESS-Plus: place of residence, race/ethnicity, occupation, gender, religion, education, socioeconomic status, social capital, plus additional factors
