# Supplementary material for: Co-Designing Person- and Family-Centered Care for Older Adults Living With HIV: Protocol for a Community-Based Participatory Study
Source: JMIR Res Protoc. 2026 Jul 10;15:e94520. doi: 10.2196/94520 (PMC13353407; doi:10.2196/94520)
Supplement: Peer Review Report 1 [file resprot-v15-e94520-s001.pdf]

**Review Type / Type d'évaluation:**	Reviewer 1 / Évaluateur 1
**Name of Applicant / Nom du chercheur:**	Kokorelias, Kristina
**Application No. / Numéro de demande:**	540979
**Agency / Agence:**	CIHR/IRSC
**Competition / Concours:**	Project Grant/Subvention Projet
**Committee / Comité:**	Health Services Evaluation & Interventions Research 3/Recherche en interventions et en évaluation dans les services de santé 3
**Title / Titre:**	Developing a Person- and Family-Centered Care Model for Older Adults Living with HIV and their Family and Chosen Family Caregivers

Adjudication Criteria/Critères de sélection

Initial Score/Cote Initiale: 4.1

Top/Bottom Selection/Groupe supérieur/inférieur

- (●) **Top/Groupe supérieur**
- ( ) **Bottom/Groupe inférieur**

**Review Type / Type d'évaluation:**	Reviewer 1 / Évaluateur 1
**Name of Applicant / Nom du chercheur:**	Kokorelias, Kristina
**Application No. / Numéro de demande:**	540979
**Agency / Agence:**	CIHR/IRSC
**Competition / Concours:**	Project Grant/Subvention Projet
**Committee / Comité:**	Health Services Evaluation & Interventions Research 3/Recherche en interventions et en évaluation dans les services de santé 3
**Title / Titre:**	Developing a Person- and Family-Centered Care Model for Older Adults Living with HIV and their Family and Chosen Family Caregivers

Summary of Application/Résumé de la demande:

The project uses an approach focused on the holistic needs of patients and caregivers.

Using a multi-method, equity-based approach, the project aims to co-design an innovative Canadian model of person- and family-centered care for older people living with HIV and their caregivers.

Qualitative data collected through interviews and co-design workshops will enable to co-create and refine a Person- and Family-Centered Care (PFCC) model for older adults living with HIV and their caregivers.

**Review Type / Type d'évaluation:**	Reviewer 1 / Évaluateur 1
**Name of Applicant / Nom du chercheur:**	Kokorelias, Kristina
**Application No. / Numéro de demande:**	540979
**Agency / Agence:**	CIHR/IRSC
**Competition / Concours:**	Project Grant/Subvention Projet
**Committee / Comité:**	Health Services Evaluation & Interventions Research 3/Recherche en interventions et en évaluation dans les services de santé 3
**Title / Titre:**	Developing a Person- and Family-Centered Care Model for Older Adults Living with HIV and their Family and Chosen Family Caregivers

Strengths and Weaknesses/Forces et faiblesses:

Significance and Impact of the Research

The project is socially and professionally relevant, as it will result in a new model adapted to the needs of older people living with HIV and their carers, particularly sex- and gender-diverse carers.

The project will have a concrete impact on improving the quality of care and support systems for older people living with HIV.

I do believe that this project can have interesting potential benefits for patients, relatives and care staff, but the benefits described are ambitious.

Approaches and Methods

The research team uses a experience-based co-design and a patient-oriented approach. The importance of transferring knowledge into practice is one of the project's strengths.

A multi-method approach including experience-based co-design, informed by previous research projects, and the Sex and Gender-Based Analysis Plus (SGBA+) framework is used.

The research team includes an advisory committee already working with them. This committee is made up of three not-for-profit administrators, two older people with HIV and five clinicians. Their collaboration plan includes quarterly meetings for the duration of the project to provide input on study activities, interpretation of data analysis and to support integrated knowledge translation.

The project's strength lies in its participatory approach, focused on the needs of individuals and the healthcare system.

The UK Design Council's Double Diamond framework is used as a guide to innovation development. Although the “Deliver” phase is integrated into the project, the researcher is limited to developing the implementation strategy and identifying barriers to implementation. This phase also includes fine-tuning and refining the model. An implementation project should ideally include a pilot in its final phase. A small pilot phase would allow concrete identification of facilitators and barriers to implementation, as well as study of the implementation process for wider implementation. The implementation process is not considered.

The selection criteria are precise, and a purposive sampling matrix will be used to ensure diverse representation by stratifying participants according to key demographic factors. Recruitment will ensure representation of both chosen and family caregivers. The heterogeneity of the participants encourages the diversity of people encountered in practice, promoting the transferability and application of the model.

Recruitment strategies are not very detailed. The researcher anticipates few recruitment difficulties thanks to existing partnerships. However, I do raise a recruitment issue when I consider the recruitment of elderly people living with HIV, thus frail elderly people. The length of interviews and workshops could be an issue for this population.

The process and structure of the workshops is very well detailed. The research team ensures pan-Ontario participation with diverse geographic representation. The project aims to produce Canada's first-ever PFCC model for older adults living with HIV, their families and selected family caregivers. However, only the province of Ontario is involved. Other Canadian provinces could have been included at this stage of the research program.

**Review Type / Type d'évaluation:**	Reviewer 1 / Évaluateur 1
**Name of Applicant / Nom du chercheur:**	Kokorelias, Kristina
**Application No. / Numéro de demande:**	540979
**Agency / Agence:**	CIHR/IRSC
**Competition / Concours:**	Project Grant/Subvention Projet
**Committee / Comité:**	Health Services Evaluation & Interventions Research 3/Recherche en interventions et en évaluation dans les services de santé 3
**Title / Titre:**	Developing a Person- and Family-Centered Care Model for Older Adults Living with HIV and their Family and Chosen Family Caregivers

Expertise, Experience and Resources

Dr. K. Kokorelias is an early career researcher with experience in qualitative research on older people living with HIV.

The research team brings together a wide range of complementary expertise to achieve the project's objectives.

The research team has the support of several organizations and individuals concerned by the subject of interest.

**Review Type / Type d'évaluation:**	Reviewer 1 / Évaluateur 1
**Name of Applicant / Nom du chercheur:**	Kokorelias, Kristina
**Application No. / Numéro de demande:**	540979
**Agency / Agence:**	CIHR/IRSC
**Competition / Concours:**	Project Grant/Subvention Projet
**Committee / Comité:**	Health Services Evaluation & Interventions Research 3/Recherche en interventions et en évaluation dans les services de santé 3
**Title / Titre:**	Developing a Person- and Family-Centered Care Model for Older Adults Living with HIV and their Family and Chosen Family Caregivers

Budget Recommendation/Recommandation budgétaire:

3 years 480 000$

Budget accepted as is.

The funding requested is part of the researcher's research program. The project and budget requested are in line with and complementary to current research work.

The budget is realistic. It includes compensation for graduates and non-graduates, as well as compensation for participants.

Please indicate your appraisal of the integration of sex as a biological variable as a strength, weakness, or not applicable to the proposal./Prière de sélectionner une option pour donner votre évaluation de l'intégration du sexe comme variable biologique en tant que point fort ou point faible de la proposition, ou en tant qu'élément non applicable à la proposition.

- ◉ **Strength/Point fort**
- ◯ **Weakness/Point faible**
- ◯ **Not applicable/Non applicable**

Please indicate your appraisal of the integration of gender as a socio-cultural determinant of health as a strength, weakness, or not applicable to the proposal./Prière de sélectionner une option pour donner votre évaluation de l'intégration du genre comme déterminant socioculturel de la santé en tant que point fort ou point faible de la proposition, ou en tant qu'élément non applicable à la proposition.

- ◉ **Strength/Point fort**
- ◯ **Weakness/Point faible**
- ◯ **Not applicable/Non applicable**

Sex and/or Gender Considerations/Notions de sexe et/ou de genre:

The project includes the use of the SGBA+ framework and the notion of intersectionality. The intersectionality perspective in analyzing data to examine how overlapping gender identities affect caregivers' roles and challenges is a strength. The development of a model to support caregivers in improving health outcomes and reducing discrimination, by promoting a more equitable and inclusive healthcare system is rooted in gender and sex-related concerns.

The development of the model takes into account the diversity of care structures, intersectional identities and systemic inequalities.

Consideration of intersectionality, in particular sexual and gender diversity, is a strength of this project.

The CCRCMA is also mobilized to ensure that the study is methodologically sound and culturally safe, and that it fosters trust between participants from diverse ethnic and linguistic backgrounds.

**Review Type / Type d'évaluation:**	Reviewer 2 / Évaluateur 2
**Name of Applicant / Nom du chercheur:**	Kokorelias, Kristina
**Application No. / Numéro de demande:**	540979
**Agency / Agence:**	CIHR/IRSC
**Competition / Concours:**	Project Grant/Subvention Projet
**Committee / Comité:**	Health Services Evaluation & Interventions Research 3/Recherche en interventions et en évaluation dans les services de santé 3
**Title / Titre:**	Developing a Person- and Family-Centered Care Model for Older Adults Living with HIV and their Family and Chosen Family Caregivers

Adjudication Criteria/Critères de sélection

Initial Score/Cote Initiale: 4.1

Top/Bottom Selection/Groupe supérieur/inférieur

- (●) **Top/Groupe supérieur**
- ( ) **Bottom/Groupe inférieur**

**Review Type / Type d'évaluation:**	Reviewer 2 / Évaluateur 2
**Name of Applicant / Nom du chercheur:**	Kokorelias, Kristina
**Application No. / Numéro de demande:**	540979
**Agency / Agence:**	CIHR/IRSC
**Competition / Concours:**	Project Grant/Subvention Projet
**Committee / Comité:**	Health Services Evaluation & Interventions Research 3/Recherche en interventions et en évaluation dans les services de santé 3
**Title / Titre:**	Developing a Person- and Family-Centered Care Model for Older Adults Living with HIV and their Family and Chosen Family Caregivers

Summary of Application/Résumé de la demande:

The project’s objective is to co-design a patient- and family-centred care (PFCC) model tailored to the needs of diverse caregivers of older adults living with HIV. It aims to center the experiences of older adults and their chosen family caregivers, explore healthcare providers' (HCPs) perspectives, and co-create both the PFCC model and an implementation strategy to improve access to health and social care in Ontario. Using a multi-method approach, the study will combine EBCD and the SGBA+ framework. It builds on prior participatory research and involves a pre-grant advisory committee comprising older adults living with HIV, clinicians, and non-profit administrators.

Multi-phase:
- Phase I: Discovering and Defining the Landscape of Care (Months 0–12): qualitative study with semi-structured interviews in ~15 older adults, 15 caregivers, 15 HCPs
- Phase II: Developing a solution: PFCC Components (Months 13–24): Co-design 2 3-h workshops using a participatory action research approach, also with 45 participants
- Phase III – Planning for Delivery (Months 25–30) by co-creating an implementation strategy for the PFCC model. ~30 participants (~10 older adults, ~10 caregivers and ~10 HCPs) across 3-4 virtual focus groups

**Review Type / Type d'évaluation:**	Reviewer 2 / Évaluateur 2
**Name of Applicant / Nom du chercheur:**	Kokorelias, Kristina
**Application No. / Numéro de demande:**	540979
**Agency / Agence:**	CIHR/IRSC
**Competition / Concours:**	Project Grant/Subvention Projet
**Committee / Comité:**	Health Services Evaluation & Interventions Research 3/Recherche en interventions et en évaluation dans les services de santé 3
**Title / Titre:**	Developing a Person- and Family-Centered Care Model for Older Adults Living with HIV and their Family and Chosen Family Caregivers

Strengths and Weaknesses/Forces et faiblesses:

Strenghts:

- This project addresses an urgent and timely topic, filling a critical research and practice gap with a strong foundation in equity, diversity, and inclusion (EDI) principles, and holds significant potential for policy impact and scalability beyond Ontario.
- The project meaningfully integrates the voices of older adults living with HIV, their caregivers, and HCPs through co-design and participatory action research. Lived experience and patient expertise are embedded at every stage, enhancing relevance and acceptability.
- The project uses established, credible methodologies (e.g., EBCD), participatory action research, and frameworks like CFIR, ensuring rigor and transparency.
- Each phase is logically connected (needs assessment co-design implementation planning), improving coherence and feasibility.
- Strategies to help ensure sustained engagement throughout the project like continuous feedback, relationship-building, and demonstrating participants' impact are included.

Weaknesses:

- While virtual delivery enhances reach, it assumes consistent digital literacy and access, which may still pose challenges for some marginalized participants, particularly older adults or low-income individuals, despite mitigation efforts.
- While facilitation is discussed, strategies for managing serious conflicts, power dynamics beyond facilitation techniques, or participant trauma responses during emotionally intense sessions are not extensively detailed.
- Recruiting and maintaining engagement with ~45 participants across two vulnerable populations (older adults with HIV and their caregivers) plus HCPs could prove difficult, even with strong partnerships. The length of interviews/workshops could be an issue for this population.
- While Phase III focuses on refining the PFCC model and planning for delivery, there is no dedicated pilot testing or feasibility study phase to validate the prototype model with participants before broader implementation. So when they wrote about "Expected Outcomes: By the end of Phase III, the research team will have finalized a refined PFCC model alongside a contextually grounded implementation roadmap, ensuring its feasibility, sustainability, and transformative potential within the existing Ontario healthcare system"- do not think this will be possible.
- In Phase 3, there is an ambition to explore scaling the model beyond HIV care to other caregiving contexts, but this could dilute focus and stretch resources without additional scoping and support.
- KT Plan: No mention of whether community partners will receive support/resources to build their KT capacity, which could enhance sustainability and equity of impact.

**Review Type / Type d'évaluation:**	Reviewer 2 / Évaluateur 2
**Name of Applicant / Nom du chercheur:**	Kokorelias, Kristina
**Application No. / Numéro de demande:**	540979
**Agency / Agence:**	CIHR/IRSC
**Competition / Concours:**	Project Grant/Subvention Projet
**Committee / Comité:**	Health Services Evaluation & Interventions Research 3/Recherche en interventions et en évaluation dans les services de santé 3
**Title / Titre:**	Developing a Person- and Family-Centered Care Model for Older Adults Living with HIV and their Family and Chosen Family Caregivers

Budget Recommendation/Recommandation budgétaire:

No concerns.

**Review Type / Type d'évaluation:**	Reviewer 2 / Évaluateur 2
**Name of Applicant / Nom du chercheur:**	Kokorelias, Kristina
**Application No. / Numéro de demande:**	540979
**Agency / Agence:**	CIHR/IRSC
**Competition / Concours:**	Project Grant/Subvention Projet
**Committee / Comité:**	Health Services Evaluation & Interventions Research 3/Recherche en interventions et en évaluation dans les services de santé 3
**Title / Titre:**	Developing a Person- and Family-Centered Care Model for Older Adults Living with HIV and their Family and Chosen Family Caregivers

Please indicate your appraisal of the integration of sex as a biological variable as a strength, weakness, or not applicable to the proposal./Prière de sélectionner une option pour donner votre évaluation de l'intégration du sexe comme variable biologique en tant que point fort ou point faible de la proposition, ou en tant qu'élément non applicable à la proposition.

- (●) **Strength/Point fort**
- ( ) **Weakness/Point faible**
- ( ) **Not applicable/Non applicable**

Please indicate your appraisal of the integration of gender as a socio-cultural determinant of health as a strength, weakness, or not applicable to the proposal./Prière de sélectionner une option pour donner votre évaluation de l'intégration du genre comme déterminant socioculturel de la santé en tant que point fort ou point faible de la proposition, ou en tant qu'élément non applicable à la proposition.

- (●) **Strength/Point fort**
- ( ) **Weakness/Point faible**
- ( ) **Not applicable/Non applicable**

**Review Type / Type d'évaluation:**	Reviewer 2 / Évaluateur 2
**Name of Applicant / Nom du chercheur:**	Kokorelias, Kristina
**Application No. / Numéro de demande:**	540979
**Agency / Agence:**	CIHR/IRSC
**Competition / Concours:**	Project Grant/Subvention Projet
**Committee / Comité:**	Health Services Evaluation & Interventions Research 3/Recherche en interventions et en évaluation dans les services de santé 3
**Title / Titre:**	Developing a Person- and Family-Centered Care Model for Older Adults Living with HIV and their Family and Chosen Family Caregivers

Sex and/or Gender Considerations/Notions de sexe et/ou de genre:

The project clearly acknowledges SGBA+ and intends to apply it systematically across sampling, data collection, analysis, and interpretation. It goes beyond sex/gender to consider how other intersectional factors (race, language, Indigeneity, disability, geography) interact with sex and gender to shape experiences of aging with HIV. However, while SGBA+ is integrated into analysis plans, it is less clear how the co-design workshops themselves will directly address sex/gender differences in facilitated discussions and outputs (are there specific prompts/questions tailored to gender-based experiences?). There’s also no mention of ongoing monitoring or evaluation to ensure that SGBA+ is consistently applied across all activities (e.g., recruitment tracking by sex/gender, facilitator reflection logs on gender dynamics during workshops).

**Review Type / Type d'évaluation:**	Reviewer 3 / Évaluateur 3
**Name of Applicant / Nom du chercheur:**	Kokorelias, Kristina
**Application No. / Numéro de demande:**	540979
**Agency / Agence:**	CIHR/IRSC
**Competition / Concours:**	Project Grant/Subvention Projet
**Committee / Comité:**	Health Services Evaluation & Interventions Research 3/Recherche en interventions et en évaluation dans les services de santé 3
**Title / Titre:**	Developing a Person- and Family-Centered Care Model for Older Adults Living with HIV and their Family and Chosen Family Caregivers

Adjudication Criteria/Critères de sélection

Initial Score/Cote Initiale: 4.4

Top/Bottom Selection/Groupe supérieur/inférieur

- (●) **Top/Groupe supérieur**
- ( ) **Bottom/Groupe inférieur**

**Review Type / Type d'évaluation:**	Reviewer 3 / Évaluateur 3
**Name of Applicant / Nom du chercheur:**	Kokorelias, Kristina
**Application No. / Numéro de demande:**	540979
**Agency / Agence:**	CIHR/IRSC
**Competition / Concours:**	Project Grant/Subvention Projet
**Committee / Comité:**	Health Services Evaluation & Interventions Research 3/Recherche en interventions et en évaluation dans les services de santé 3
**Title / Titre:**	Developing a Person- and Family-Centered Care Model for Older Adults Living with HIV and their Family and Chosen Family Caregivers

Summary of Application/Résumé de la demande:

This research aims to develop the first person- and family-centered care (PFCC) model specifically designed for older adults living with HIV, including chosen family caregivers. Using a co-design approach informed by the SGBA+ framework, older adults and their caregivers will be actively engaged in four main steps (discover, define, develop, and deliver) in shaping the model to reflect their needs and experiences. The project seeks to improve healthcare experiences, strengthen caregiver support, and create a model adaptable to other regions and populations.

**Review Type / Type d'évaluation:**	Reviewer 3 / Évaluateur 3
**Name of Applicant / Nom du chercheur:**	Kokorelias, Kristina
**Application No. / Numéro de demande:**	540979
**Agency / Agence:**	CIHR/IRSC
**Competition / Concours:**	Project Grant/Subvention Projet
**Committee / Comité:**	Health Services Evaluation & Interventions Research 3/Recherche en interventions et en évaluation dans les services de santé 3
**Title / Titre:**	Developing a Person- and Family-Centered Care Model for Older Adults Living with HIV and their Family and Chosen Family Caregivers

Strengths and Weaknesses/Forces et faiblesses:

Strengths

1.Important study that addresses a critical knowledge and practice gap. Proposal is well written and very easy to follow

2.Great team. They are well positioned to conduct this research. NPI has excellent track record

3.SGBA was well integrated throughout the study

4.Ikt approaches well described. Meaningful community engagement and that of PWLE

Weaknesses

1.This is a study that involves vulnerable populations (i.e., old age, + HIV/AIDS + minority groups). Any specific strategies to address potential (real and perceived) safety, privacy, confidentiality concerns? The strategies indicated for addressing power differentials and ethical concerns are too generic.

2.They proposed to older people with limited technology competencies to organizations that provide technology access. While this seems to address access to technology, there are some potential setbacks regarding privacy, safety and stigma concerns. Would participants feel comfortable going to such places? What strategies are indicated to facilitate trust? Any prior relationship with such organizations?

3.Regarding caregiver role, looks like the study is focused on chosen family caregivers as defined in the proposal but recruitment seems to be focused on “care givers” as defined in the proposal. Isn’t this taking the focus away from the intended population? Shouldn’t the chosen family givers be recruited?

**Review Type / Type d'évaluation:**	Reviewer 3 / Évaluateur 3
**Name of Applicant / Nom du chercheur:**	Kokorelias, Kristina
**Application No. / Numéro de demande:**	540979
**Agency / Agence:**	CIHR/IRSC
**Competition / Concours:**	Project Grant/Subvention Projet
**Committee / Comité:**	Health Services Evaluation & Interventions Research 3/Recherche en interventions et en évaluation dans les services de santé 3
**Title / Titre:**	Developing a Person- and Family-Centered Care Model for Older Adults Living with HIV and their Family and Chosen Family Caregivers

Budget Recommendation/Recommandation budgétaire:

No concerns

Please indicate your appraisal of the integration of sex as a biological variable as a strength, weakness, or not applicable to the proposal./Prière de sélectionner une option pour donner votre évaluation de l'intégration du sexe comme variable biologique en tant que point fort ou point faible de la proposition, ou en tant qu'élément non applicable à la proposition.

- ◉ **Strength/Point fort**
- ◯ **Weakness/Point faible**
- ◯ **Not applicable/Non applicable**

Please indicate your appraisal of the integration of gender as a socio-cultural determinant of health as a strength, weakness, or not applicable to the proposal./Prière de sélectionner une option pour donner votre évaluation de l'intégration du genre comme déterminant socioculturel de la santé en tant que point fort ou point faible de la proposition, ou en tant qu'élément non applicable à la proposition.

- ◉ **Strength/Point fort**
- ◯ **Weakness/Point faible**
- ◯ **Not applicable/Non applicable**

**Review Type / Type d'évaluation:**	Reviewer 3 / Évaluateur 3
**Name of Applicant / Nom du chercheur:**	Kokorelias, Kristina
**Application No. / Numéro de demande:**	540979
**Agency / Agence:**	CIHR/IRSC
**Competition / Concours:**	Project Grant/Subvention Projet
**Committee / Comité:**	Health Services Evaluation & Interventions Research 3/Recherche en interventions et en évaluation dans les services de santé 3
**Title / Titre:**	Developing a Person- and Family-Centered Care Model for Older Adults Living with HIV and their Family and Chosen Family Caregivers

Sex and/or Gender Considerations/Notions de sexe et/ou de genre:
